# A new parasitoid wasp, *Aphaereta vondelparkensis* sp. n. (Braconidae, Alysiinae), from a city park in the centre of Amsterdam

**DOI:** 10.3897/BDJ.8.e49017

**Published:** 2020-01-27

**Authors:** Kees van Achterberg, Menno Schilthuizen, Marrit van der Meer, Richard Delval, Claudia Dias, Marlene Hoynck, Heko Köster, Rudie Maarschall, Norbert Peeters, Peter Venema, Ryan Zaremba, Cristina Beltrami, Florinda Nieuwenhuis, Nicole de Rop, Iva Njunjić, Joris M. Koene

**Affiliations:** 1 Naturalis, Leiden, Netherlands Naturalis Leiden Netherlands; 2 Zhejiang University, Hangzhou, China Zhejiang University Hangzhou China; 3 Taxon Expeditions, Leiden, Netherlands Taxon Expeditions Leiden Netherlands; 4 Naturalis Biodiversity Center, Leiden, Netherlands Naturalis Biodiversity Center Leiden Netherlands; 5 Francis W. Parker School, Chicago, United States of America Francis W. Parker School Chicago United States of America; 6 University of Verona, Verona, Italy University of Verona Verona Italy; 7 Gemeente Amsterdam, Amsterdam, Netherlands Gemeente Amsterdam Amsterdam Netherlands; 8 Vrije Universiteit, Amsterdam, Netherlands Vrije Universiteit Amsterdam Netherlands

**Keywords:** Alysiini, new species, Palaearctic, Netherlands, endoparasitoid, dipterous larvae, decaying meat

## Abstract

**Background:**

The parasitic wasp genus *Aphaereta* Foerster, 1863, belongs to the large subfamily Alysiinae Leach, 1815 (Hymenoptera: Braconidae) and contains solitary or gregarious endoparasitoids of larvae of cyclorrhaphous Diptera living in decaying organic matter.

**New information:**

A new species, *Aphaereta vondelparkensis* sp. n. (Braconidae, Alysiinae, Alysiini), from a city park in the centre of Amsterdam (Vondelpark) is described and illustrated. The species was discovered on a so-called taxon expedition, in which experts and citizen scientists collaborate on taxonomic projects. The finding highlights the fact that new animal species can still be discovered today, even in the busiest park of the capital of one of the best studied European countries.

## Introduction

*Aphaereta* Foerster, 1863, belongs to the large subfamily Alysiinae Leach, 1815 (Hymenoptera: Braconidae) and contains solitary or gregarious endoparasitoids of larvae of cyclorrhaphous Diptera living in decaying organic matter belonging mainly to the families Anthomyiidae, Calliphoridae, Drosophilidae, Fanniidae, Muscidae, Sciomyzidae, Sepsidae, Tachinidae and Tephritidae. The genus has a cosmopolitan distribution and contains 45 valid species (Yu et al. 2016). During a citizen science ‘Taxon Expedition’ (for the term see Schilthuizen et al. (2017) and http://www.taxonexpeditions.com) in the oldest park of Amsterdam (Vondelpark, opened in 1865), an undescribed species of *Aphaereta* was found. The holotype of *A. vondelparkensis* was collected in a trap baited with decaying meat and its biology is uncertain, but paratypes were reared as parasitoids of Calliphoridae and Sarcophagidae larvae living on decaying carcasses.

## Materials and methods

The collecting site of the holotype was the Koeienweide in the Vondelpark in the centre of Amsterdam (52.3557°N 4.8588°E) and the collecting was done with a pitfall trap baited with decaying chicken meat. Additional specimens were reared from a dead rabbit in the dunes of Oostvoorne (Zuid Holland) with *Parasarcophaga caerulescens* Zett. (Calliphoridae) larvae. Specimens were collected and stored in 70% ethanol and subsequently prepared according to the AXA method (van Achterberg 2009, van Achterberg et al. 2010) and glued on card points. Images were acquired with a KEYENCE VHX-5000 Digital Microscope imaging system and processed with Photoshop CS5 software. Terminology follows van Achterberg (1988). RMNH stands for Naturalis Biodiversity Center, Leiden.

## Taxon treatments

### Aphaereta vondelparkensis sp. n.

94FC15C4-D5BA-5071-B16C-275DE8AFD712

urn:lsid:zoobank.org:act:0D788CC9-BE51-4293-BC0C-C85C2C170506

#### Materials

- **Type status:** Holotype. **Occurrence:** recordedBy: Taxon Expeditions participants; **Taxon:** family: Braconidae; genus: Aphaereta; specificEpithet: vondelparkensis; scientificNameAuthorship: van Achterberg et al.; **Location:** continent: Europe; country: The Netherlands; municipality: Amsterdam; locality: Vondelpark: Koeienweide; **Event:** samplingProtocol: bait-trap with meat; year: 2019; month: 07; day: 07; **Record Level:** institutionID: RMNH
- **Type status:** Paratype. **Occurrence:** recordedBy: J. Huijbregts; sex: 7 females; **Location:** continent: Europe; country: The Netherlands; municipality: Oostvoorne; locality: Quackjeswater; **Event:** samplingProtocol: from dead rabbit with *Parasarcophaga caerulescens* Zett. (Sarcoph.); year: 2014; month: 08; day: 30; eventRemarks: ex pupa *Parasarcophaga caerulescens* (Zett.), det. L. Sijstermans 2014; **Record Level:** institutionCode: RMNH; basisOfRecord: 7 preserved specimens
- **Type status:** Paratype. **Occurrence:** sex: 2 females; **Taxon:** family: Braconidae; genus: Aphaereta; specificEpithet: vondelparkensis; **Location:** continent: Europe; country: The Netherlands; municipality: Oostvoorne; locality: Quackjeswater; **Event:** samplingProtocol: culture in lab., M. Lammers, RMNH’16; year: 2014; month: 10; day: 15; eventRemarks: Ex *Calliphora vomitoria* (Calliphoridae) in lab., F1-2, em. 15.x.2014, greg. par., M. Lammers, RMNH’16; **Record Level:** institutionID: RMNH; institutionCode: RMNH; basisOfRecord: PreservedSpecimen
- **Type status:** Paratype. **Occurrence:** sex: 1 males; **Taxon:** family: Braconidae; genus: Aphaereta; specificEpithet: vondelparkensis; **Location:** continent: Europe; country: The Netherlands; municipality: Oostvoorne; locality: Quackjeswater; **Event:** samplingProtocol: culture in lab., M. Lammers, RMNH’16; year: 2014; month: 10; day: 17; eventRemarks: Ex *Calliphora vomitoria* (Calliphoridae) in lab., F1-2, em. 17.x.2014, greg. par., M. Lammers, RMNH’16; **Record Level:** institutionID: RMNH; institutionCode: RMNH; basisOfRecord: PreservedSpecimen
- **Type status:** Paratype. **Occurrence:** sex: 3 females; **Taxon:** family: Braconidae; genus: Aphaereta; specificEpithet: vondelparkensis; **Location:** continent: Europe; country: The Netherlands; municipality: Oostvoorne; locality: Quackjeswater; **Event:** samplingProtocol: culture in lab., M. Lammers, RMNH’16; year: 2014; month: 10; day: 21; eventRemarks: Ex *Calliphora vomitoria* (Calliphoridae) in lab., F1-2, em. 21.x.2014, greg. par., M. Lammers, RMNH’16; **Record Level:** institutionID: RMNH; institutionCode: RMNH; basisOfRecord: PreservedSpecimen
- **Type status:** Paratype. **Occurrence:** sex: 8 females; **Taxon:** family: Braconidae; genus: Aphaereta; specificEpithet: vondelparkensis; **Location:** continent: Europe; country: The Netherlands; municipality: Oostvoorne; locality: Quackjeswater; **Event:** samplingProtocol: culture in lab., M. Lammers, RMNH’16; year: 2014; month: 10; day: 27; eventRemarks: Ex *Calliphora vomitoria* (Calliphoridae) in lab., F1-2, em. 27.x.2014, greg. par., M. Lammers, RMNH’16; **Record Level:** institutionID: RMNH, ZJUH; institutionCode: RMNH, ZJUH; basisOfRecord: PreservedSpecimen
- **Type status:** Paratype. **Occurrence:** sex: 1 female; **Taxon:** family: Braconidae; genus: Aphaereta; specificEpithet: vondelparkensis; **Location:** continent: Europe; country: The Netherlands; municipality: Oostvoorne; locality: Quackjeswater; **Event:** samplingProtocol: culture in lab., M. Lammers, RMNH’16; year: 2014; month: 09; day: 18-22; eventRemarks: Ex *Calliphora vomitoria* (Calliphoridae) in lab., F1-2, em. 18-22.ix.2014, greg. par., M. Lammers, RMNH’16; **Record Level:** institutionID: RMNH; institutionCode: RMNH; basisOfRecord: PreservedSpecimen
- **Type status:** Paratype. **Occurrence:** recordedBy: P. Mayhew; sex: 3 females, 1 male; **Taxon:** family: Braconidae; genus: Aphaereta; specificEpithet: vondelparkensis; **Location:** continent: Europe; country: The Netherlands; municipality: Leiden; **Event:** samplingProtocol: ex liver baits (gregarious [parasitoid]); year: 1977; month: 06; eventRemarks: RMNH’97; **Record Level:** institutionID: RMNH; institutionCode: RMNH; basisOfRecord: PreservedSpecimen

#### Description

Holotype, ♀, length of body 1.8 mm, of fore wing 2.0 mm.

**Figure 1.**
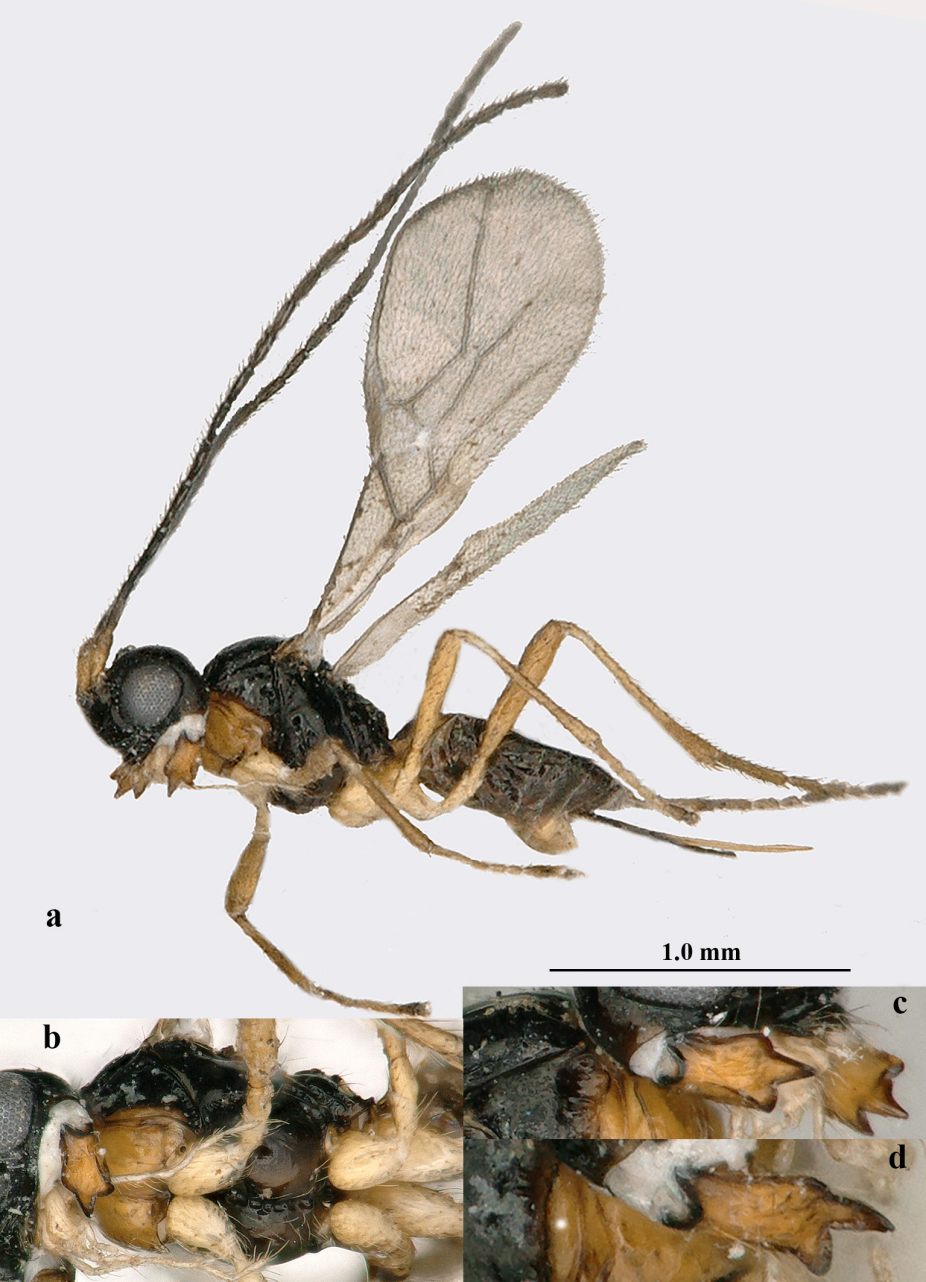
*Aphaereta vondelparkensis* sp. n., ♀, holotype. **a.** habitus, lateral aspect; **b.** mesosoma, ventral aspect; **c.** mandible, full sight on middle tooth; **d.** id., but full sight on third tooth.

**Figure 2.**
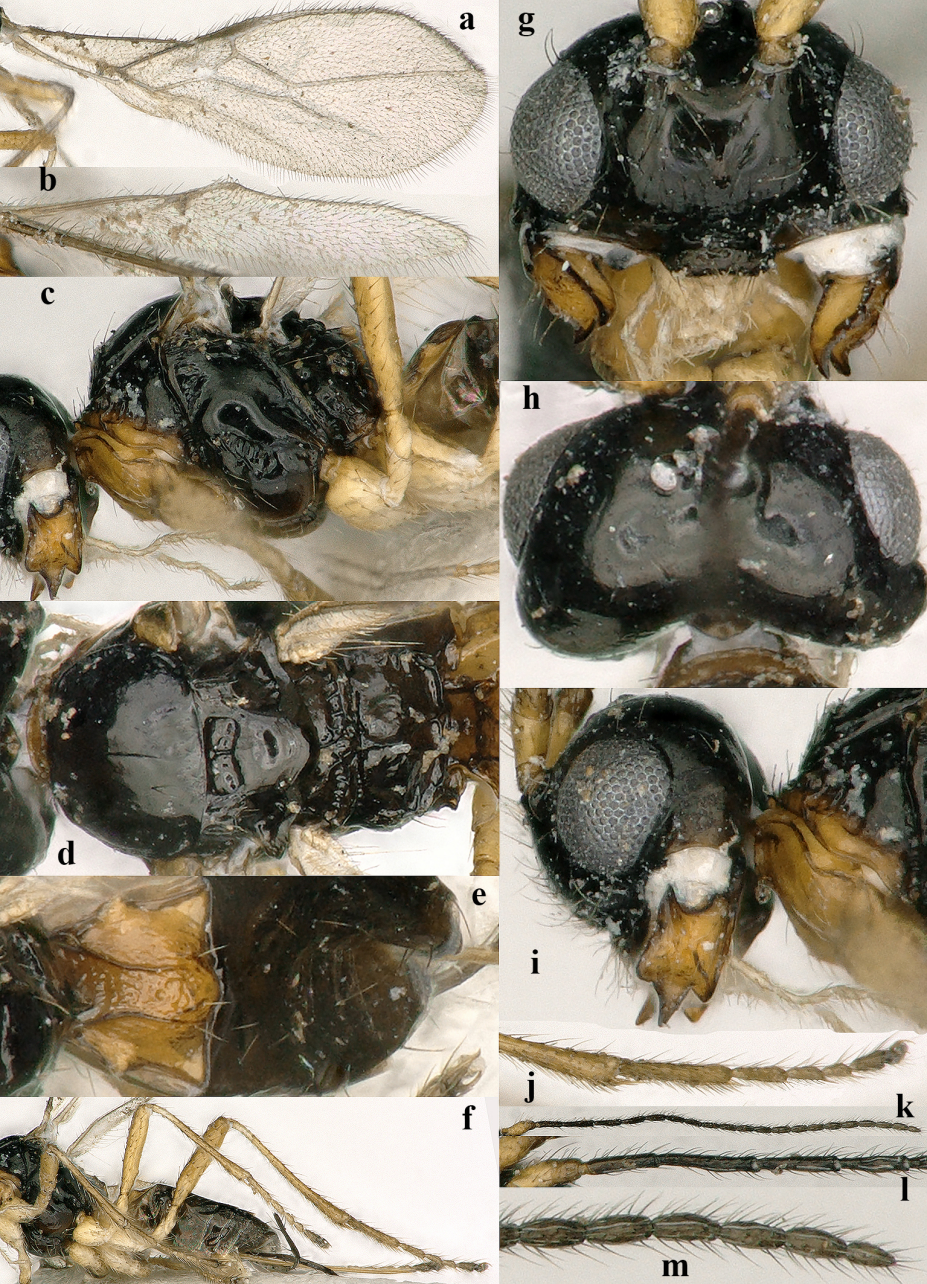
*Aphaereta vondelparkensis* sp. n., ♀, holotype. **a.** fore wing; **b.** hind wing; **c.** mesosoma, lateral aspect; **d.** mesosoma, dorsal aspect; **e.** first metasomal tergite, dorsal aspect; **f.** legs; **g.** head, anterior aspect; **h.** head, dorsal aspect; **i.** head, lateral aspect; **j.** hind tarsus, lateral aspect; **k.** antenna; **l.** base of antennae, lateral aspect; **m.** apex of antenna.

*Head.* Antenna 1.4 times length of fore wing and 1.6 times body (Fig. 1a), with 21 segments, setae erect and about 1.3 times as long as width of segment, length of third segment 0.8 times fourth segment (Fig. 2l), length of third, fourth and penultimate segments 5.0, 6.3 and 3.0 times their maximum width, respectively; fourth segment narrowed basally in lateral view (Fig. 2l); maxillary and labial palp with 6 and 4 segments, respectively; length of maxillary palp 1.2 times height of head; OOL:diameter of posterior ocellus:POL = 7:2:3; frons glabrous and smooth, nearly flat; length of eye in dorsal view 1.6 times temple (Fig. 2h); eyes glabrous except for a few setae; temples nearly parallel-sided behind eyes (Fig. 2h); median groove of vertex shallow; face 1.5 times wider than high medially, smooth, but medio-ventrally somewhat rugulose, with long erect upward directed setae; clypeus largely smooth, sparsely finely punctate, rather convex medially, long setose, not tuberculate laterally and ventral rim truncate (Fig. 2g); epistomal groove rather wide, laterally smooth and distinct (Fig. 2g); anterior tentorial pits medium-sized (Fig. 2g); length of malar space 0.2 times basal width of mandible; mandible medially rugulose, hardly widened subapically, its medial length 1.5 times maximum width, second tooth much longer than both lobe-shaped lateral teeth, without incision between first and second tooth, third tooth with curved carina (Fig. 1a–d); head 1.6 times as wide as mesoscutum (Fig. 2d).

*Mesosoma.* Length of mesosoma 1.3 times its height; pronope absent, but posteriorly with a transverse and indistinctly crenulate groove and no antescutal depression; side of pronotum largely smooth, antero-medially and ventro-posteriorly finely crenulate (Fig. 2c), ventral area moderately convex, medium-sized and yellowish-orange; precoxal sulcus entirely impressed, but only anteriorly and medially moderately crenulate (Fig. 2c); mesosternal sulcus medium-sized and moderately crenulate (Fig. 1b); metapleuron smooth dorsally and medially, rugose ventrally; notauli absent, only anteriorly with horizontal carina (Fig. 2d); medio-posterior depression of mesoscutum absent; mesoscutum glabrous (except for a few setae), smooth, strongly shiny and with complete lateral carina; scutellar sulcus medium-sized, transverse and deep, with one long and two short crenulae; axillar depression narrow, smooth and rather shallow (Fig. 2d); scutellum slightly convex; anteriorly metanotum with short median carina and not protruding dorsally (Fig. 2c); surface of propodeum mainly smooth, without lateral protuberance, its medio-longitudinal carina distinctly lamelliform and protuberant and posteriorly with narrow triangular medial area (Fig. 2c,d).

*Wings.* Fore wing: 2-SR oblique; r:3-SR:SR1 = 4:20:57; r vertical and about as long as width of pterostigma, narrow (about 4 times as long as its own width), distance between base of pterostigma and base of vein r 2.6 times as long as vein r and distinctly angled with 2-SR (Fig. 2a); disco-marginal cell slender (Fig. 2a; pterostigma narrow elliptical; SR1 straight and ending nearly at apex of wing; 1-CU1:2-CU1 = 1:9; 2-SR:3-SR:r-m = 11:20:3; r-m oblique; first subdiscal cell open ventrally and apically; CU1a at level of 2-CU1; m-cu slightly converging to 1-M. Hind wing: narrow apically; no trace of cu-a and m-cu; basal cell open posteriorly and veins largely hardly or not sclerotised (Fig. 2b).

*Legs.* Hind coxa smooth; tarsal claws very slender (Fig. 2f and j), no distinct protuberance but widened basally; middle femur slender and parallel-sided; middle tibia moderately erect setose (Fig. 2f); fourth segment of middle tarsus 1.5 times longer than wide in dorsal view and bristly setose and bristles somewhat shorter than twice width of segment; length of femur, tibia and basitarsus of hind leg 5.4, 10.1 and 6.0 times their width, respectively; hind femur largely smooth and parallel-sided, but basally narrowed; hind tibia with erect setae, somewhat widened apically and subbasally setose; hind tarsal segments with rather short and sparsely setose ventro-apical protuberance (Fig. 2j); hind basitarsus comparatively slender and parallel-sided, apically with medium-sized acute lamella (Fig. 2j); length of hind tibial spurs 0.20 and 0.25 times hind basitarsus.

*Metasoma.* Length of first tergite equal to its apical width, its surface distinctly convex medially and finely rugose medially, remainder largely smooth (Fig. 2e), its dorsal carinae distinct in basal 0.9 and tergite widened behind spiracles (Fig. 2e); dorsope rather large; second tergite smooth; ovipositor straight; length of setose part of ovipositor sheath 0.37 times fore wing, equal to metasoma, 1.6 times hind femur and 1.1 times hind tibia, with very long setae, ribbon-shaped (except apically) and with a short apical spine; hypopygium medium-sized and apically subtruncate (Fig. 1a).

*Colour.* Black or dark brown; pedicellus of antenna brown; palpi, tegulum, coxae, trochanters, trochantelli and metasoma largely medio-ventrally (including hypopygium) pale yellowish; scapus and remainder of legs (but telotarsi infuscate) brownish-yellow; humeral plate and remainder of antenna dark brown; mandible (but margins darkened), propleuron, pronotal side ventrally and first tergite orange; pterostigma, parastigma and vein r brown; wing membrane slightly infuscate.

*Variation.* Length of fore wing 1.8–2.9 mm; antenna of ♀ with 21(2), 22(2), 23(3), 24(3), 25(9) or 26(6) segments; third antennal segment 0.6–0.8 times as long as fourth segment; metasoma ventrally often largely dark brown and only hypopygium yellowish; third antennal segment of ♀ usually slender (Fig. 2l), but sometimes robust, 3.8–5.0 times longer than wide; second submarginal cell of fore wing slender in small specimens (Fig. 2a) to robust in larger specimens, 2.5–2.8 times longer than its maximum width; vein 2-SR of fore wing 2.4–2.8 times as long as vein r; vein 1+2-CU1 of fore wing distinctly pigmented in nearly all specimens; propodeal areola present, narrow triangular or medium-sized to narrow parallel-sided and dorsally distinctly protruding. The only male examined is very similar to females and it has 27 antennal segments.

#### Etymology

Named after the famous city park of Amsterdam (Vondelpark) where the holotype was collected during the Taxon Expedition in Amsterdam.

#### Distribution

Netherlands.

#### Biology

Gregarious parasitoid of Sarcophagidae and Calliphoridae larvae living in decaying carcasses.

## Discussion

The new species belongs to the *Aphaereta minuta* group (as defined by Oliveira et al. (2012)) and runs to *A. minuta* (Nees, 1811), also because some specimens have 21 or 22 antennal segments (19–22 segments in *A. minuta*), convex ventral area of pronotal side wide, vein r of fore wing emitted before middle of pterostigma and vein r-m of fore wing usually vertical. It differs from *A. minuta* by having the first tergite paler than second tergite and orange or yellowish-brown (tergites similarly coloured and first tergite dark to pale brown in *A. minuta*) and lateral smooth and flattened areas wider than medial convex and sculptured area (narrower), third and fourth segments of hind tarsus medium-sized and basitarsus similarly coloured as hind femur (slender and basitarsus often darker than hind femur), distance between base of pterostigma and base of vein r approx. 2.5 times as long as vein r (thrice as long), medio-longitudinal propodeal carina in lateral view distinctly protruding (not protruding), the setose part of ovipositor sheath about 1.1 times as long as hind tibia (about 0.8 times) and the pedicellus brown, darker than scapus (more or less yellowish dorsally and similar to scapus). Similar species with yellowish or orange first tergite (e.g. *A. pallipes* (Say, 1829)) have the distance between base of pterostigma and base of vein r 3 times as long as vein r (2.6 times in *A. vondelparkensis* sp. n.; Fig. 2a), hind wing with closed cell (partly open because of reduced (only pigmented) veins 1r-m and M+CU&1-M; Fig. 2b) and vein r-m of fore wing inclivous (vertical or nearly so in *A. vondelparkensis* sp. n.; Fig. 2a). The new species runs in the key by Fischer (1966) to the Nearctic *A. pallipes* (Say, 1829) if the colour of the first tergite is used (the second and only other character (the relative length of the third antennal segment; 0.6–0.7 times fourth segment) is too variable in the reared series to be useful), but this species has the carinae of the propodeum less protruding, the costulae at least partly present dorsally and the first tergite is slightly more slender (1.1–1.2 times longer than its apical width). Wharton (1977) restricted in his analysis of the New World species *A. pallipes* to populations associated with fresh cow manure, which, according to his synoptic table, have the ovipositor [sheath] distinctly longer than 1.5 times the hind tibia. Both biology and relative length of ovipositor sheath disagree with *A. vondelparkensis* sp. n. according to Wharton (1977). There is a synonym of *A. pallipes* described from Sarcophagidae (*A. sarcophagae* Gahan, 1914), but it was reared from a parasitoid of Orthoptera (Blaesoxipha (Kellymyia) kellyi (Aldrich, 1914) and has vein 2-SR of fore wing twice as long as vein r (Gahan 1914; 2.4–2.8 times in *A. vondelparkensis* sp. n.). The Nearctic *A. genevensis* Fischer, 1966 is a species very similar to *A. pallipes* (Wharton 1977), but it is a solitary species with less antennal segments (19–20 in ♀), first and second metasomal tergites similarly reddish coloured and ovipositor sheath shorter than hind tibia (Fischer 1966). In the key to Afrotropical species by Peris-Felipo (2015), it runs to *A. basirufa* Granger, 1949 from Madagascar. *Aphaereta basirufa* has no strongly protruding carinae medially on the propodeum (present in *A. vondelparkensis* sp. n.), face twice as wide as high medially (1.5 times), no propodeal areola (present), vein r emitted near middle of pterostigma (slightly before middle) and hind tarsus conspicuously densely erect setose (moderately setose).

The fact that we were able to discover a new species of insect in the busiest urban park in the capital city of the Netherlands, one of the biologically best-studied countries of the world, highlights how little we still know of large sections of biodiversity. It is therefore especially fortunate that members of the general public were involved in the discovery and description of this species.

## Supplementary Material

XML Treatment for Aphaereta vondelparkensis
