# Corrigendum to "A new parasitoid wasp, *Aphaereta vondelparkensis* sp. n. (Braconidae, Alysiinae), from a city park in the centre of Amsterdam"

**DOI:** 10.3897/BDJ.8.e50971

**Published:** 2020-02-25

**Authors:** Menno Schilthuizen, Kees van Achterberg

**Affiliations:** 1 Taxon Expeditions, Leiden, Netherlands Taxon Expeditions Leiden Netherlands; 2 Naturalis Biodiversity Center, Leiden, Netherlands Naturalis Biodiversity Center Leiden Netherlands; 3 Naturalis, Leiden, Netherlands Naturalis Leiden Netherlands; 4 Zhejiang University, Hangzhou, China Zhejiang University Hangzhou China

## Abstract

This corrigendum serves to recognise the contribution of Dr. Mark Lammers, who was erroneously not given authorship in the original version of the paper.

## Statement

In a recent publication (van Achterberg et al. 2020), Dr. Mark Lammers, of the Vrije Universiteit, Amsterdam, Netherlands was erroneously withheld authorship. Dr. Lammers provided a large amount of cultured paratype material of the new species, which was used to assess the new species' variability. He also provided details on its lifecycle, which served the discussion on the biology of the new species. We hereby wish to rectify this by assigning post-publication co-authorship (in 3^rd^ position, between Schilthuizen and Van der Meer) to Dr. Lammers. This corrigendum does not, however, alter the authorship of the species itself.
